# Development and Validation of a Gender Ideology Scale for Family Planning Services in Rural China

**DOI:** 10.1371/journal.pone.0059919

**Published:** 2013-04-01

**Authors:** Xueyan Yang, Shuzhuo Li, Marcus W. Feldman

**Affiliations:** 1 School of Public Policy and Administration and Shaanxi Laboratory for Population and Development Research, Xi’an Jiaotong University, Xi’an, Shaanxi Province, People’s Republic of China; 2 Morrison Institute for Population and Resource Studies, Stanford University, Stanford, California, United States of America; Tulane University, United States of America

## Abstract

The objectives of this study are to develop a scale of gender role ideology appropriate for assessing Quality of Care in family planning services for rural China. Literature review, focus-group discussions and in-depth interviews with service providers and clients from two counties in eastern and western China, as well as experts’ assessments, were used to develop a scale for family planning services. Psychometric methodologies were applied to samples of 601 service clients and 541 service providers from a survey in a district in central China to validate its internal consistency, reliability, and construct validity with realistic and strategic dimensions. This scale is found to be reliable and valid, and has prospects for application both academically and practically in the field.

## Introduction

### Quality of Care in Family Planning and Gender Issues in Rural China

Family planning (family planning) is a core component of China’s policies for economic and social development. Since China implemented its fertility control policy in the late 1970s, its population growth has been successfully curbed, and women’s reproductive health promoted [1]. However, the policy has had a number of negative impacts on women, some of which derive from placing most responsibility for contraception on women while paying insufficient attention to women’s reproductive health [2]–[4].

In order to reduce the negative effects of family planning and to respond to the Program of Action of the International Conference on Population and Development and the Beijing Declaration of the Fourth World Conference on Women, the National Population and Family Planning Commission of China (NPFPC) in 1995 launched a pilot program to improve Quality of Care in family planning services that was centered on the needs of service clients. The objectives were to provide service clients with high quality and comprehensive services, such as diversified and personalized services for reproductive health, informed choice in contraception, monitoring of reproductive health, management and evaluation of family planning services [5]–[7].

Achievements in Quality of Care over the past ten years were documented both in the field visit evaluation reports by the national Quality of Care office and articles and reports by scholars and experts. These articles and reports addressed two issues: first, whether the reproductive rights of service clients could be protected without generating a higher birth rate, which had been a source of concern for some; second, whether the quality of family planning work has improved and reproductive health of service clients enhanced [8], [9]. There was also discussion of deficiencies in Quality of Care, especially concerning gender issues and it was pointed out that not enough attention had been paid to gender issues in Quality of Care in family planning services. This has obstructed further improvements of the Quality of Care in family planning services [8]–[11]. It was claimed that this occurred mostly because people working in family planning services failed to understand gender issues, i.e., that they were “lacking gender ideology”. “Gender ideology” is the common abbreviation for “equitable gender role ideology” or “equitable gender role attitude,” which includes a positive attitude towards equity of men’s and women’s gender roles [8]–[12].

In rural China, the Quality of Care in family planning services involves two kinds of people: one is “service providers,” namely the family planning workers including decision makers, administrators, and technical service providers at county, township and village levels. The other is “service clients” whose definition varies with the development of the Quality of Care in family planning services. In the past, “service clients” has referred only to married women of childbearing ages who were treated as birth control targets. Integrating a gender perspective into the Quality of Care would enable family planning service to include clients at various life-course stages and of different genders, such as men of various ages, married women of child-bearing ages, unmarried women, menopausal and post-menopausal women, youth and adolescents [13]. However, gender mainstreaming in family planning services in rural China urgently requires adequate instruments to measure gender role ideology of family planning service providers and clients. Appropriate advocacy and interventions can then be introduced to integrate gender perspectives into the Quality of Care in family planning services.

### The Existing Research on Gender Role Ideology

Since the 1930s, researchers in Western countries have developed many scales to measure concepts related to gender role ideology. Among these are the sex-role belief scale (SRBS) [14], the sex-role ideology scale (SRIS) [15], the attitudes toward women scale (AWS) [16], and the sex role egalitarianism scale (SRES) [17]. There is variability in the operational definition of and factors included in these gender roles. Details of these scales are shown in Table 1.

**Table 1:** Gender Role Ideology from Various Scales.

Scale	Underlying Concept	Construct	Authors
SRBS	Beliefs and attitudes toward the roles that maleand female should play in society	Single dimension	Kerr & Holden
SRIS	Ideology may be described as liberal, modern, feminist or egalitarian on the one hand, or as traditional orconservative on the other hand	At least two dimensions (traditionalism and feminism) are included in practical application	Kalin, Heusser & Edwards
AWS	Attitudes toward female’s role, rights andresponsibilities	Three dimensions: attitudes towards female roles, female rights, and female responsibilities	Spence & Helmreich
SRES	Attitudes towards the traditional and nontraditionalroles played by males and females in marriage, birth,employment, inter-personal and inter-sex relationship, education	Including two orders (formal and informal relationship) and five dimensions (marriage, reproduction, employment, inter-personal and inter-sex relationship, education)	King, et al. 1997

In different cultures, gender role ideology has been related to different issues, but many scholars have adopted similar terms to describe its dimensions: one is modern, i.e., feminist, with belief in equality; another is traditional, i.e., sexist, with belief in the acceptability of inequality. Accordingly, gender role ideology is a two-dimensional concept [18].

Cross-cultural, cross-national, and cross-regional differences in gender construction have meant that scales developed earlier are not directly applicable to the cultural background of China. In addition, the subjects studied with most attitude scales have been limited to college students, feminists, career women, and housewives, who are not representative of the target participants of our study, namely the service clients and providers of family planning services at the grass-roots level in China. Further, the contents of these scales reflect gender role ideology in such fields as family life, politics, leadership, education, raising children, and employment, and while some might be related to reproductive health, they cannot cover the full extent of the family planning field [18], [19]. Thus, there is a lack of measurement instruments and scales applicable to the family planning field in rural China.

The objective of this study is to develop a gender ideology scale for family planning (GIS-family planning) that is applicable to the Quality of Care in family planning and reproductive health services in rural areas of China, and which can provide a reliable and valid measurement tool for the study of gender role ideology of service clients and providers, and consequently to help develop strategies and measures that incorporate a gender perspective into family planning services in rural China.

## Methods

### Concept Definition on Gender Role Ideology

Most rural areas in China are characterized by the Han culture, which has patrilineality, patrilocality, and patriarchy as its core, and in which male preference exists in land and resource allocation, while in reproduction the culture of son preference is dominant [8], [20]. Before Quality of Care became a goal of family planning, China’s family panning policy had birth control as its goal, and administrative measures were implemented to achieve this goal. For instance, one-child or two daughter families were rewarded by the government, and those families with more births than the national plan allows were punished with fines (or so-called social maintenance fee). These measures reduced the opportunities for reproductive choices, interacted with the traditional reproductive culture, further strengthened son preference in reproductive ideology, and influenced people’s attitudes towards gender roles in all sectors of family planning [21]. With respect to contraception and birth control, most service clients, even some service providers thought that birth was a woman’s matter and contraception was a woman’s responsibility, not a concern of men. Reproduction was for the multiplication of the family; therefore, the whole family’s interest should be taken into consideration in deciding whether the woman should use contraception, and regarding what measures should be used; the husband, father-in-law and mother-in-law should make decisions for a woman with respect to contraceptive use. Inequality between men and women was accepted and it was believed that men should be dominant and controlling [22]. With respect to family planning, those service clients and providers thought that procreation and reproduction were relevant only to married women of childbearing age, and family planning was irrelevant to men, menopausal and post-menopausal women, unmarried women, and adolescents and youth [23], [24]. For the management of family planning services, it was accepted that birth control was the top priority, and married women of childbearing age were the means to control population size and growth. Thus the needs and rights of reproductive age women should be subservient to the goal of birth control [4], [11], [25], [26].

As Quality of Care became an important goal of family planning services, cooperation with international programs has been increased, migration of a large fraction of the rural population has been occurred, and women’s status has improved, new ideas have begun to be introduced into the family planning field in rural China. There has been a new understanding of men and women’s roles in family planning, and recognition of the need for men’s participation in contraception and birth control [27]–[29]. However, broad understanding of gender issues in Quality of Care for family planning services is still lacking. People commonly treat gender equity in family planning as males’ participation in family planning and shared responsibility for contraception while ignoring that people of different genders and ages deserve the rights and benefits of family planning, such as informed choice of contraceptive use and diversified and personalized Quality of Care. In recent years, some experts in gender issues in the family planning field of China have promoted gender equity among service providers and service clients by means of advocacy and training, etc., but the deeply rooted traditional reproductive culture formed over millennia cannot be changed quickly [8]–[10].

A famous book on social psychology by Penner defined attitude as a concept with three components including cognitive, affective and behavioral intention parts [30]. Therefore, based on the academic and practical background described above, according to the three components of attitude defined by Penner, here we define the concept of “gender ideology for family planning” as “affective attitude with a realistic ideology dimension and a strategic ideology dimension”; this is, therefore, a two-dimensional concept.

The meanings of “realistic” and “strategic” are extensions of the related concepts in the gender needs analysis framework of the Development Planning Unit of London University (DPU) [31]. The DPU defined women’s gender needs as a concept with realistic and strategic dimensions. The realistic needs are “the needs which are in relation to women’s realistic living, such as demands for clean water, health care etc. Satisfying such needs could not change the existing unequal gender roles”; the strategic needs are “the needs which are related to women’s strategic activities, such as demands for political participation, education etc. Satisfying such needs could change the existing traditional unequal gender roles, and build more equal gender relations.”

In the present study, realistic ideology refers to “an attitude towards the relations between males and females under the traditional division of gender roles”; in the family planning field, it is defined as “an attitude towards male and female roles defined by traditional reproductive culture” [7], [32]. Strategic ideology refers to “an advocacy that challenges the traditional relations between genders and also wants to alter the traditional partition of roles and the dominance relationship between male and female” [31]; in the family planning field it is denial of the traditional division of gender roles and advocacy of breaking the traditional restrictions on male and female roles in family planning, while establishing a new and more equitable gender relationship [7], [32].

### Items Pool

The contents of items in existing scales were modified to form new items. We emphasized: (1) Contents related to marriage, reproduction, sexuality, reproductive health or family planning in previous scales of gender role attitudes, including the sex-role egalitarianism scale developed by Beere, King, Beere & King [33], the sex-role ideology scale developed in North America by Kalin et al. [15], and the gender role belief scale developed by Kerr & Holden [14]. (2) Contents related to gender role attitudes in reproductive health or family planning attitude questionnaires, including attitudes towards contraception, and Quality of Care in family planning services [34]–[37].

Items 1, 2, 19, 20 were generated from literature review as shown in Table 2.

**Table 2:** Development of GIS-family planning.

Item	Originally Proposed 34 Items	Experts’ Assessed 26 Items	Statistically Validated 15 Items
**Realistic Ideology**			
1 Contraception should only be women’s responsibility, and have nothing to do with men	**√**	**√**	**√**
2 Gynecological diseases are women’s problems, and should not involve men	**√**	**√**	**√**
3 The wife should be the first to be medically examined when a couple cannot procreate	**√**	**√**	
4 Women should not make excessive demands of their husbands in sexual life	**√**	**√**	**√**
5 It is shameful for menopausal women to consult about sexual issues	**√**	**√**	**√**
6 Abortion services should not be provided for unmarried pregnant women, as it encourages immoral behaviors	**√**	**√**	
7 Doctors’ discriminatory attitudes against unmarried pregnant girls in medical treatment are acceptable	**√**	**√**	**√**
8 It is worth paying the social maintenance fee to give birth to a boy outside the birth quota	**√**	**√**	**√**
9 It is not worth paying the social maintenance fee to give birth to a girl outside the birth quota	**√**	**√**	**√**
10 In order to prevent transmission and spread of STDs, female STD patients’ personal informationshould be open to the public	**√**	**√**	**√**
11 A wife who wants to have abortion should first get permission from her husband	**√**	**√**	
12 Villages should be allowed to release information on women’s use of contraceptives to betterassist family planning	**√**	**√**	
13 Villages should be allowed to release information on men’s sterilization to better assistfamily planning	**√**	**√**	
14 It is not necessary to provide sexual knowledge to adolescents, as they will get to know it eventually.	**√**		
15 When choosing contraceptives, childbearing women should listen to suggestions fromservice providers	**√**		
16 Men should listen to suggestions from service providers, concerning whether to usecontraceptives and which to use	**√**		
17 It is acceptable that a family planning institution can only meet the needs of the majority of clients	**√**		
18 It is important for clients to get free condoms; whether condoms are comfortableis a secondary consideration	**√**		
**Strategic Ideology**			
19 Men should actively take responsibilities for contraception	**√**	**√**	
20 A husband should be cooperative and supportive when his wife receives treatment forgynecological diseases such as reproductive tract diseases	**√**	**√**	**√**
21 The husband should accompany his wife when she receives an induced abortion	**√**	**√**	**√**
22 The husband should be cooperative and supportive to help his wife deal with menopausal problems	**√**	**√**	**√**
23 The wife should help her husband enjoy a satisfying sexual life	**√**	**√**	
24 A wife should be considerate of her husband when he has a disorder of sexual function dueto pressure from work or other things	**√**	**√**	**√**
25 The husband should help his wife enjoy a satisfying sexual life	**√**	**√**	**√**
26 Men should acquire as much sexual knowledge as they can	**√**	**√**	**√**
27 Women should acquire as much sexual knowledge as they can	**√**	**√**	**√**
28 It is not shameful for a man to see a doctor because of diseases of the reproductive orurological systems	**√**	**√**	
29 Women should have the right to decide whether to abort a pregnancy	**√**	**√**	
30 Contraceptives should be conveniently available and accessible to unmarried peopleregardless of their gender	**√**	**√**	
31 Privacy of all clients, no matter what their gender or ages are (including those withvenereal diseases and sex workers), should be protected	**√**	**√**	
32 Family planning institutions should meet the needs of childbearing women	**√**		
33 Family planning institutions should meet the needs of men	**√**		
34 Family planning institutions should meet the needs of all clients regardless of their gender or ages	**√**		

Note: Each item has a 5-point Likert response: (1) strongly agree (2) agree (3) neither agree nor disagree (4) disagree (5) strongly disagree.

Two groups of interviews were conducted to generate other new items. We selected Hancheng county of Shaanxi province for the first interview in April 2004. Hancheng is a national project county for Quality of Care in family planning services in Shaanxi province. It has strong son preference, and an underdeveloped economy. We assumed that the realistic ideology might be dominant in Hancheng. Secondly, we selected Deqing county of Zhejiang province for the second interview in June 2004. Deqing is a national project county for Quality of Care in family planning services in Zhejiang province. It has a well-developed economy, and weak son preference. We assumed that the strategic ideology might be dominant in Deqing.

In each county we first conducted two focus-group discussions with service providers at the county level, including one group of administrators with six interviewees, and one group of technical service providers with six interviewees; three in-depth interviews of service providers at the county level, including one decision-maker, one administrator, and one technical service provider.

Second, we selected two townships according to their performances in family planning work, including a relatively good one and one that was not so good. In each township, we conducted two focus-group discussions with service providers at the township level, including one group of administrators with four interviewees, one group of technical service providers with four interviewees, and three in-depth interviews of service providers at the township level, including one decision-maker, one administrator, and one technical service provider.

Third, we selected one village with medium performance in family planning work from each of the two townships mentioned above. For each village, there is only one family planning worker who is simultaneously responsible for administration and technical services, so we conducted only one in-depth interview with the family planning worker. Additionally, we conducted two focus-group discussions with service clients, respectively, with men and women, each including eight married interviewees aged from 20 to 49 years. We also carried out two in-depth interviews, respectively, with one unmarried man about 20 years old and one unmarried woman about 20 years old.

The interviews mainly concerned: what women should and should not do in family planning? What men should and should not do in family planning? What work have service providers done for men as service clients with respect to diversified and personalized services regarding reproductive health, informed choice in contraception, monitoring of reproductive health, management and evaluation of family planning services? What have service providers done for women as service clients? The qualitative data were used to generate items to measure gender role attitudes in family planning services as reflected by service providers at various levels of family planning institutions, and service clients of different genders and different life-course stages.

The grounded theory was used to analyze the data from the above interviews and to form items that reflect attitudes towards gender roles in family planning in rural China. Items 3–18 and 21–34 were generated from the above interviews as shown in Table 2.

Finally, we proposed a 34 item pool for measuring gender ideology, including 18 items on the realistic dimension and 16 items on the strategic dimension, as shown in Table 2.

### Item Selection

In order to select items to form a scale and to ensure content validity and operability of the items in the scale, we organized three experts’ assessments with different aims.

The first assessment was held in Beijing in September of 2004, with the participation of two experts on gender and one expert on Quality of Care in family planning services. The purpose was to evaluate the content validity of the scale, especially: (1) whether the concept of gender ideology is reflected by the items; (2) whether the scores can be obtained from the answers to the items.

The second assessment was held in Xi’an, Shaanxi province, in October of 2004, with two psychologists experienced in the development and application of scales. The purpose was to evaluate the operability of the scale, namely: (1) would the items in the scale be understood and answered by most participants; (2) was the structural arrangement of the scale scientific and reasonable.

The third assessment was held in Xi’an in November 2004, with one expert on scale development and three experts on questionnaire design. During this session the operability of the scale survey was appraised, including: (1) selection of the survey samples; (2) confirmation of the survey approach; (3) quality-control of the survey procedure.

As a result of the process described above, eight items were removed from the 34 item pool, namely five items on the realistic dimension, items 14, 15, 16, 17, and 18; and three items on the strategic dimension, items 32, 33, and 34. In addition, two items on the realistic dimension were revised, including item 1, namely “Gynecological diseases are a matter for women, and not a concern for men” was modified to “Contraception should only be women’s responsibility, and have nothing to do with men”, and item 7 namely “It is acceptable for doctors to show a negative judgment when they treat unmarried pregnant girls” was modified to “Doctors’ discriminatory attitudes against unmarried pregnant girls in medical treatment are acceptable”.

Finally, the result was a 26-item gender ideology scale for family planning (GIS-family planning) with thirteen negative items on the realistic dimension and thirteen positive items on the strategic dimension, as shown in Table 2. All items are randomly arranged in the questionnaire, and the higher the scores on the scale, the more equitable is the gender ideology. This 26-item scale was statistically tested and validated by empirical data in following sections.

### Samples and Data Analyses

The sample questionnaire survey to test and validate the 26-item scale was conducted in Juchao district of Chaohu city in Anhui province of central China in June 2005. Juchao is a national project district for Quality of Care in family planning services in Anhui province. It has strong son preference, and a medium-developed economy. The Institute for Population and Development Studies, Xi’an Jiaotong University, established the first “Experimental Zone for Improving Girl Child Survival” in Chaohu City with support from the Chinese government in 2000. Since then, the Institute and the governments of Chaohu city and Juchao district have established a long-term cooperative relationship [38]. Juchao district is a mostly rural area with a large number of rural-urban migrants and, as such, is not only a representative area where both traditional and equitable gender role ideologies are prevalent, but also an appropriate location for the administration of our complicated survey.

Participants were service clients and service providers of family planning services. For service clients, we first planned to select four townships according to the family planning work, including a better one, two medium ones and a worse one; second, from each township, we planned to randomly select five villages; third, from each village, we planned to randomly select thirty service clients according to gender and age, including five men and women from each of the three age groups 20 to 29 years, 30 to 39 years, and 40 to 49 years. In total, the survey was designed to achieve a sample size of 600 service clients, including 300 men and 300 women. The local coordinators from each village also assisted us in convening all respondents to gather in the village’s administration office. For these service clients, we carried out an interview survey of those who were illiterate or had difficulty reading, while those who could read filled out a questionnaire independently. In implementing the survey, when we had difficulty in convening or interviewing respondents, samples were slightly adjusted based on suggestions of local coordinators. As a social science project rather than a medical or biological experiment, this study is not subject to the law of “ethical examination on bio-medical study involving human beings” made by Health Ministry of China (see item three chapter one). During the procedure, all participants provided their informed consent statements. When the participants were illiterate, we read the consent statements to them, and with the illiterates’ consents, their friends or acquaintances signed the illiterates’ names on the informed consent statements. We finally obtained 601 valid samples of service clients, including 306 men and 295 women. Of these, most were married (97.8 percent) and aged from 20 to 49 years, with a few unmarried people (2.2 percent).

For service providers, we first requested that all service providers at the district and township levels (22 townships) should be included in the survey; second, we planned to randomly select 10 to15 service providers at the village level from all villages under each township’s jurisdiction. In total, the survey included about 500 service providers. We also requested that all service providers at the district level gather in the district’s administration office, and all service providers at the township level and at its jurisdictional village level gather in the township’s administration office. We conducted a questionnaire survey of these service providers with instructions necessary to ensure the quality of the whole survey. We finally obtained 541 valid samples of service providers, including 5.1 percent at the district level, 37.2 percent at the township level, and 57.7 percent at the village level. Of these, 116 were men and 425 were women.

Item analysis and factor analysis were used to select items and finalize the scale; reliability analysis and validity analysis were conducted to measure and validate the quality of the final scale.

Item analysis computed the ratio of the mean scores of each item in the high-scoring group (respondents with the highest 25 percent of scores) and the low-scoring group (respondents with the lowest 25 percent of scores). This is called the critical ratio (CR), and CR above 3 indicates acceptable discriminability of a scale item. Those items with CR below 3 were regarded as having poor discriminability and were subsequently removed.

Principal component analysis was used to abstract the factors, and direct oblimin method was used to rotate axis. According to a scree plot output and the assumptions behind the concept construction of gender ideology, the number of the factors abstracted was restricted to two, and items whose loading was lower than 0.3 on both dimensions were removed.

Alpha internal consistency coefficients on the total scale and sub-scales among total samples and sub-samples were calculated to assess the reliability of the final scale, and structural equation modeling (SEM) was used to assess the validity of the final scale. Construct validity includes convergent validity and discriminant validity. Convergent validity was assessed with confirmatory factor analysis (CFA), on the assumption that the gender ideology concept includes realistic and strategic dimensions, from which the indices with close to or higher than threshold values indicate satisfactory convergent validity. Here we proposed three models for validation: a one-factor model, an uncorrelated two-factor model, and a correlated two-factor model. Following the suggestions of Anderson & Gerbing [39], discriminant validity was tested by conducting two separate CFAs. The correlation coefficient between the two factors in one CFA was defined as 1, but was undefined in the other. From each CFA, we obtained a correspondingΧ2; and the difference between the twoΧ2 is denoted △Χ2 The significance of △Χ2 indicates whether discriminant validity is satisfactory.

## Results and Discussion

### Item Analysis

Item analysis and factor analysis were applied to samples to determine items to be removed from the 26 item scale. 11 items including items 3, 6, 11, 12 and 13 on the realistic dimension and items 19, 23, 28, 29, 30 and 31 on the strategic dimension were removed due to either their poor discriminability or low loading. As a result, we obtained the final 15-item GIS-family planning with eight negative items on the realistic dimension and seven positive items on the strategic dimension, as shown in Table 2.

### Reliability Analysis

Reliability analysis was conducted on the 15-item GIS-family planning. Scores of GIS-family planning ranged from 28 to 75 with a mean of 57 and standard deviation 6.06. In Table 3, the alpha coefficients of internal consistency of the 15-item GIS-family planning are shown. The alpha coefficients in the total sample and sub-samples of the total GIS-family planning were mostly (80 percent) higher than 0.7 and all the alpha coefficients of subscales were higher than 0.65, which suggests acceptable reliability of GIS-family planning.

**Table 3:** Alpha Internal Consistency Coefficients for 15-item GIS-family planning.

GIS-family planning	Men (422)	Women (720)	Service Clients (601)	Service Providers (541)	Total Sample (1142)
Realistic dimension	0.72	0.73	0.68	0.69	0.73
Strategic dimension	0.74	0.71	0.71	0.73	0.73
Total scale (15 items)	0.75	0.75	0.70	0.75	0.75

### Validity Analysis

A one-factor model, an uncorrelated two-factor model, and a correlated two-factor model were tested on the 15-item GIS-family planning. The results suggest that the correlated two-factor model fitted the data better than the others, and the correlation coefficient between the two factors is 0.40. The factor loadings for the correlated two-factor model are shown in Table 4, and the results for CFA are shown in Table 5.

**Table 4:** Factor Loadings for 15-item GIS-family planning.

Item	M	SD	r	Loadings	
**Realistic Ideology**					
Item 1	3.60	0.95	0.43	0.52***	
Item 2	4.02	0.78	0.47	0.44***	
Item 4	3.28	0.98	0.40	0.50**	
Item 5	3.72	0.82	0.44	0.45***	
Item 7	3.47	1.05	0.37	0.49***	
Item 8	3.70	1.07	0.39	0.52***	
Item 9	3.72	0.99	0.39	0.51***	
Item 10	3.15	1.22	0.30	0.50**	
**Strategic Ideology**					
Item 20	4.22	0.72	0.35		0.33***
Item 21	4.11	0.71	0.34		0.38***
Item 22	4.16	0.57	0.40		0.31***
Item 24	4.13	0.62	0.30		0.30***
Item 25	3.89	0.68	0.28		0.30***
Item 26	4.02	0.63	0.31		0.39***
Item 27	3.99	0.66	0.25		0.38***

Note: t>1.96,

* p<0.05; t>2.58,

** p<0.01; t>3.29,

*** p<0.001; M is mean score, SD is Standard Deviation, r is corrected item-total correlations.

**Table 5:** Fit of Indices of CFA for 15-item GIS-family planning.

GIS-family planning	*Χ*2/df	RMSEA	GFI	PGFI	NFI	IFI	CFI
One-factor model	16.96	0.12	0.85	0.64	0.62	0.64	0.64
Uncorrelated two-factor model	7.30	0.071	0.93	0.70	0.81	0.83	0.83
Correlated two-factor model	6.83	0.064	0.94	0.70	0.84	0.87	0.87

Note: Χ2/df is the Normal Minimum Fit Function Chi-Square divided by Degrees of Freedom with threshold less than 2; RMSEA is Root Mean Square Error of Approximation with threshold less than 0.06; GFI is Goodness of Fit Index with threshold above 0.90, and PGFI is Parsimony Goodness of Fit Index with threshold above 0.50; NFI is Normed Fit Index with threshold above 0.90; IFI is Incremental Fit Index with threshold above 0.90; CFI is Comparative Fit Index with threshold above 0.90.

The results from the convergent validity analysis suggested that the best fitting model (correlated two-factor model) should be tested for discriminant validity. Good discriminant validity is confirmed, as shown in Table 6. Thus, results from the various statistical analyses above have demonstrated satisfactory quality of the final 15-item GIS-family planning.

**Table 6:** Discriminant Validity for 15-item GIS-family planning.

	*Χ*2	df
Undefined model	509.69	89
Defined model	1526.54	90
△Χ2	1016.85***	

Note: △Χ2 is the difference between theΧ2 of the defined model with CFA (in which the correlation coefficient between the two factors was defined as 1) and undefined model with CFA (in which the correlation coefficient between the two factors was undefined); △Χ2>2.71,

+ p<0.1; △Χ2>3.84,

* p<0.05;△Χ2>6.64,

** p<0.01; △Χ2>10.83,

*** p<0.001.

### Conclusions

In this study, a scale of gender ideology for family planning, namely GIS-family planning, was constructed for measuring gender equity of service clients’ and service providers’ attitudes toward men’s and women’s roles in the family planning domain in rural China. The psychometric properties of the scale were examined using survey data from Juchao district in Anhui province of central China.

Our data analysis indicates acceptable reliability for GIS-family planning (0.75), and both convergent validity and discriminant validity for GIS-family planning were satisfactory. The results from CFA support the construct assumptions, with “gender ideology for family planning” composed of two correlated factors, including realistic ideology and strategic ideology. We conclude that GIS-family planning is a reliable and valid measure of gender equity ideology for service clients and providers of family planning services in rural China. The scale may be applied in two areas. One is academic; the scale could be regarded as an independent variable for further study of gender ideology of service clients and service providers for family planning; or as a predictor for studying the impact of gender ideology for family planning on individual behaviors of service clients and providers. The second is practical; the scale could be applied in assessing Quality of Care in family planning services in rural China, for evaluating the ideologies of service clients and service providers, and providing performance assessments and promotion strategies in terms of gender equity.

Limitations of our study include: (1) Limitations of the sample and data. First, among the total sample, there are some service clients who were illiterate or had difficulty reading to whom the interview survey methods were applied, and these may cause courteous biases, i.e. the tendency for respondents to give answers that they think the interviewer wants to hear, rather than what they really feel. Second, the service providers and clients usually have different educational and professional background in rural China, so using the same questionnaire for both may cause the systematic bias; finally, GIS-family planning was tested in only one district in central China. (2) Limitations of the content validity. Although the questionnaire was assessed several times by experts from various fields, there has been disagreement concerning gender ideology and, in particular, the realistic and strategic dimensions for family planning. These different viewpoints of experts were reflected in the items of the questionnaire, and may affect the validity of the items themselves.

The future study should be addressed: assess the validity and utility of the GIS-family planning in other samples from other areas, such as eastern China and western China. Make improvements to GIS-family planning by testing it on samples from other areas after modification of items.

## References

[1] Jin WJ (2010) An analysis on effects of family planning on gender equality promotion, Population and Family Planning (in Chinese). 7: 18–20.

[2] Kaufman J, Zhang ZR, Qiao XJ, Zhang Y (1992) Quality of family planning services in rural China. Studies in Family Planning 23 (2): 73–84. 1604461

[3] Kaufman J, Zhang ZR, Qiao XJ, Zhang Y (1992) The creation of family planning service stations in China. International Family Planning Perspectives, 18 (1): 18–23.

[4] Zhu CZ, Li SZ, Qiu CR, Hu P, Jin AR (1997) The dual effects of the family planning program on Chinese women (in both Chinese and English). Xi’an, China: Xi’an Jiaotong University Press, 5–10 p.

[5] Gu BC, Zhang EL, Xie ZM (1999) Toward a Quality of Care approach reorientation of the family planning program in China, In: Satia J, Patricia M, Lim AT, editors: Innovation. Kuala Lumpur. Malaysia (ICOMP) 7–8.

[6] Gu BC, Simmons R, Szatkowski D (2002) Offering a choice of contraceptives in Deqing County, China: changing practice in the family planning program since 1995, in: Haberland N, Measham D, editors: Responding to Cairo: Case Studies of Changing Practice in Reproductive Health and Family Planning. New York: Population Council, Standing Committee 16. (58–73).

[7] Zhu CZ, Yang XY, Li SZ (2004) The basic textbook of gender (in Chinese). Beijing, China: China Population Press, 18–24 p.

[8] Hardee K, Xie ZM, Gu BC (2004) Family planning and women’s lives in rural China. International Family Planning Perspectives 30 (2): 68–76. 15210405 10.1363/3006804

[9] Kaufman J, Fang J, Liu YG (2004) Baseline report: China case study on gender and health equity, Dafang County, Guizhou Province, China. Unpublished report prepared for 2004 Gender and Health Equity Network (GHEN) Coordination Meeting, Beijing. 5–10 p.

[10] Holcombe S (2003) Assessment report on the 2001–2003 phase of implementing the National Population and Family Planning Commission’s pilot project in Quality of Care. Supported by the Ford Foundation in China, 8–21 p.

[11] Liu HY (2003) The gender issues in reproductive health. Population and Family Planning (in Chinese) 9: 30–32.

[12] Yang XY, Li SZ, Wu Z, Schimmele CM (2009) Developing scales for measuring gender behaviors in reproductive health in rural China, Biodemography and Social Biology. 55 (1): 82–92. 10.1080/19485560903054721 19835102

[13] Liu C (2010) A gender analysis into the reproductive health services, Journal of China Women’s University (in Chinese). 5: 27–33.

[14] Kerr P, Holden R (1996) Development of the gender role beliefs scale. Journal of Social Behavior Personality, Special Issue 11 (5): 3–14.

[15] Kalin R, Heusser C, Edwards J (1982) Cross-national equivalence of a sex-role ideology scale. Journal of Social Psychology 116: 141–142.

[16] Spence J, Helmreich R (1972) The attitudes toward women scale: an objective instrument to measure the attitudes towards the rights and roles of Women in contemporary society. JSAS: Catalog of Selected Documents in Psychology 2: 66–67.

[17] King L, King D, Gudanowski D,Taft C (1997) Latent structure of the sex-role egalitarianism scale: confirmatory Factor Analysis. Sex Roles, 36, 221–234.

[18] Gibbons J, Hamby B, Dennis W (1997) Researching gender-role ideologies internationally and cross-culturally. Psychology of Women Quarterly 21: 151–170.

[19] McHugh M, Frieze I (1997) The measurement of gender-role attitudes. Psychology of Women Quarterly 21: 1–16.

[20] Li J (2005) Women’s status in a rural Chinese setting. Rural Sociology 70 (2): 229–252.

[21] Li JH (2004) Gender inequality, family planning, and maternal and child care in a rural Chinese county. Social Science & Medicine 59: 695–708. 15177828 10.1016/j.socscimed.2003.11.041

[22] Yen FT (2007) The impact of gender and hierarchy on women’s reproductive health in a Kaml, Village, Guizhou Province, China. Culture, Health & Sexuality 9 (1): 55–68. 10.1080/13691050600965653 17364714

[23] Kaufman J, Zhang EL, Xie ZM (2005) Family planning Quality of Care in China: scaling up a pilot project into a national reform program. in: Simmons R, Fajans P and Ghiron L, editors: From Pilot Projects to Policies and Programs: Strategies for Scaling Up Innovations in Health Service Delivery (WHO).(3–5).

[24] Kaufman J, Zhang EL, Xie ZM, Zhang KN (2006) Quality of Care in China: from pilot project to national program. Studies in Family Planning, 37 (1), 17–28. 10.1111/j.1728-4465.2006.00080.x 16570727

[25] Mu GZ (2000) Changing of Chinese conceptions since family planning policy implemented. Newspaper of Technology (in Chinese) 12: 26–29.

[26] Lu L, Li XY (2001) The comparison study between Chinese and foreign women. Journal of China Women University in Shandong Region (in Chinese) 4: 8–11.

[27] Wang C, Vittinghoff E, Lu SH, Wang HY, Zhou MR (1998) Reducing pregnancy and induced abortion rates in China: family planning with husband participation. American Journal of Public Health, 88 (4): 646–648. 10.2105/ajph.88.4.646 PMC1508427 9551010

[28] Simmons R, Diaz M (2002) Facilitating large-scale transitions to Quality of Care: an idea whose time has come. Studies in Family Planning 33 (1): 61–75. 11974420 10.1111/j.1728-4465.2002.00061.x

[29] Simmons R, Hall J, Diaz M, Fajans P, Satia J (1997) The strategic approach to contraceptive introduction. Studies in Family Planning 28 (2): 79–94. 9216029

[30] Penner L (1986) Social psychology: concepts and applications. New York, Los Angeles, San Francisco: West Publishing Company, 352–400 p.

[31] Smyth I, March C, Mukhopadhyay M (1998) A guide to gender-analysis frameworks. OXFAM. 8–10 p.

[32] Yang XY, Wu KJ, Li SZ (2005) The gender needs analysis in reproductive health: based on the gender needs analysis framework and the survey in project county of Quality of Care for family planning. Collection of Women’s Studies (in Chinese) 5: 35–39.

[33] Beere C, King D, Beere D, King L (1984) The sex-role egalitarianism scale: a measure of attitudes towards equality between the sexes. Sex Roles 10: 563–576.

[34] Sawyer SM, Phelan PD, Bowes G (1995) Reproductive health in young women with cystic fibrosis: knowledge, behavior and attitudes. Journal of Adolescent Health 17: 46–50. 7578163 10.1016/1054-139X(94)00096-W

[35] Bender S (1999) attitudes of Icelandic young people toward sexual and reproductive health. Family Planning Perspectives 31 (6): 294–301. 10614520

[36] Mogilevkina I, Tyden T, Odlind V (2000) Ukrainian medical students’ experiences, attitudes, and knowledge about reproductive health. Journal of American College Health 49 (6): 269–272. 10.1080/07448480109596313 11413944

[37] Psihogios V, Rodda C, Reid E, Clark M, Clarke C, et al (2002) Reproductive health in individuals with homozygous β-thalassemia: knowledge, attitudes, and behavior. Fertility and Sterility 77 (1): 119–127. 11779601 10.1016/s0015-0282(01)02933-8

[38] Hvistendahl M (2009) Making every baby girl count. Science 323 (27): 1164–1166. 19251606 10.1126/science.323.5918.1164

[39] Anderson J, Gerbing D (1988) Structural equation modeling in practice, a review and recommended two-step approach. Psychological Bulletin 103 (3): 411–423.

